# Addressing disparities in academic medicine: what of the minority tax?

**DOI:** 10.1186/s12909-015-0290-9

**Published:** 2015-02-01

**Authors:** José E Rodríguez, Kendall M Campbell, Linda H Pololi

**Affiliations:** 1 The Center for Underrepresented Minorities in Academic Medicine at The Florida State University College of Medicine, 1115 West Call Street#3210 M, Tallahassee, FL 32306 USA; 2 Women’s Studies Research Center, Mailstop 079, Waltham, MA 02454-9110 USA

**Keywords:** Underrepresented minority, Black, Latino, Hispanic, Native american, Minority tax

## Abstract

**Background:**

The proportion of black, Latino, and Native American faculty in U.S. academic medical centers has remained almost unchanged over the last 20 years. Some authors credit the "minority tax"—the burden of extra responsibilities placed on minority faculty in the name of diversity. This tax is in reality very complex, and a major source of inequity in academic medicine.

**Discussion:**

The “minority tax” is better described as an Underrepresented Minority in Medicine (URMM) faculty responsibility disparity. This disparity is evident in many areas: diversity efforts, racism, isolation, mentorship, clinical responsibilities, and promotion.

**Summary:**

The authors examine the components of the URMM responsibility disparity and use information from the medical literature and from human resources to suggest practical steps that can be taken by academic leaders and policymakers to move toward establishing faculty equity and thus increase the numbers of black, Latino, and Native American faculty in academic medicine.

## Background

The proportion of black, Latino, and Native American faculty in U.S. academic medical centers increased slightly over the last 20 years (7% vs. 8%) [1] Multiple medical organizations, including the American Medical Association (AMA), the American Association of Medical Colleges (AAMC) and the National Medical Association (NMA) have been working to increase the representation of those in racial/ethnic groups that are underrepresented in medicine. Although progress has been made in increasing the numbers of medical students and faculty from URMM backgrounds, the proportions of URMM faculty and URMM students remain basically unchanged since the numbers of positions has increased. The unchanged proportion is far below the targets set by the AMA, the AAMC and the NMA.

Our review of the literature identified factors that affect minority faculty in academic medicine. These factors have been colloquially called the “minority tax” or “cultural tax”. The minority tax has been defined as the tax of extra responsibilities placed on minority faculty in the name of efforts to achieve diversity [2,3]—but this unfair tax is, in reality, complex. For the purposes of this article, we will focus on those who are Underrepresented Minorities in Medicine (URMM), including blacks, Latinos, and Native Americans/Alaskans. Other minorities, particularly Asians, may not be underrepresented in medicine, but still suffer many, if not all, of the disparities addressed. Unlike taxes, (which are theoretically shared by all) the following responsibilities are not shared equally by all faculty, and disproportionately burden URMM faculty. The URMM faculty responsibility “tax” or disparity includes the following categories: responsibility for achieving diversity efforts, racism, isolation, mentorship, clinical, and promotion inequities.

## Discussion

### Diversity efforts disparity

Many underrepresented URMM faculty feel an obligation to the communities they represent and to future generations of minority students [2]. As a result, they choose to spend more of their time working in community efforts, and they are often asked to take on committee work in the area of diversity [4]. URMM faculty are disproportionately represented in institutions’ diversity efforts, illustrating the disparity that exists in this area. These diversity-related pursuits have been devalued in some institutions, and not taken seriously as promotion-earning activities [4]. Importantly, these efforts are time consuming and result in URMM faculty having less time to engage in pursuits that are more valued by their institutions. Some URMM faculty find that this extra work presents a conflict of interest because the institution’s espoused goals for diversity and care of the underserved are not aligned with the reality of what is rewarded and supported by the organization [4]. In one study, URMM faculty in institutions where the majority of faculty were from URMM groups felt more values alignment than URMM faculty in traditional medical schools [5].

### Racism disparity

Numerous studies of URMM faculty have reported racism, discrimination and inequity in academic medicine as a major problem [2,4-8]. Racism has been cited as a major cause of job dissatisfaction. URMM faculty have gone so far as to publish responses to discrimination [6,8,9]. URMM faculty also feel that there is racism or bias in the promotions process [8], a course of action that seems to favor non-URMM faculty over URMM faculty [2]. Racism has also been named as a reason URMM faculty feel that they have to constantly prove their value, worth and ability [6], thus diverting their energies from more meaningful activities. URMM faculty agree that although racism has become subtler [4,6,7], it remains harmful and can have lasting effects. URMM faculty have to expend more effort to combat racism than their non-URMM peers as they have not only to defend themselves against racism but also take more responsibility for righting the injustice of racism generally.

### Isolation disparity

URMM faculty feel excluded [10], invisible [4,6,11], isolated [5], and of poor fit [11,12]. They report that a sense of belonging to the institution is essential for success [6] and career satisfaction. They also feel the isolation derives in part, from the fact that there are few or no other persons in their departments or institutions that look like them [4]. This isolation additionally impacts minority faculty by limiting opportunity for collaboration and scholarly activity.

Stereotypic thinking by colleagues acts as a barrier to potential collaboration. For example, URMM faculty are often viewed as lacking skills in literature review, research, writing and publication, or URMM faculty are sometimes seen as similar to “uneducated minority patients or other people of color in service roles”. [4] Often the expertise of URMM faculty members is not fully recognized by non-URMM colleagues, and URMM are not readily identified as valuable collaborators. This is unfortunate as diverse perspectives enrich research collaboration and would enhance outcomes, and benefit health care and the institution.

### Mentorship disparity

URMM faculty believe that having mentors and role models is crucial for their success [2,8,10,11,13,14]. URMM faculty at individual institutions and in national samples express that there are inadequate numbers of mentors for URMM faculty [15]. Mentors are indispensable in helping URMM faculty feel that they belong [2] to the academic institution and to help them navigate the complex process of academic promotion and tenure. The absence of mentors may contribute to URMM underrepresentation in academic medicine [4,6]. Functional mentoring relationships are also associated with career satisfaction [2].

URMM faculty also serve as role models for URMM students and house staff, and act as mentors for them. URMM faculty thus become mentors without the benefit of having mentoring and guidance for themselves. Non-URMM faculty need to receive training in mentoring URMM faculty and students, to help alleviate this disparity. Mentoring is a teachable skill.

### Clinical disparity

This aspect of the tax is subtler than other contributing factors. URMM faculty spend more of their time in community work [4] and caring for underserved populations [5], and clinical activities [7,16,17] than non-URMM faculty. There is an inverse relationship between clinical time and scholarly productivity: as clinical time increases, time for scholarly productivity decreases, resulting in less time for promotion related activities. This may contribute to the presence of fewer URMM faculty in senior positions, such as full professor or department chair [18]. URMM faculty tend to care for poorer patients [18], making their clinical revenues less than those of their peers. This further disadvantages URMM faculty with respect to promotion since they have not only less time for scholarship but also need more time for clinical care to generate revenue comparable to their non-URMM counterparts.

### Promotion disparity

It is well documented that a promotion disparity exists between URMM and non-URMM faculty [16,19,20]. URMM faculty are more frequently found in junior faculty positions than leadership positions [1]. They are promoted at lower rates than their non-URMM counterparts [21] (Table 1). Spending more time on diversity efforts and in clinical activities, lacking effective mentors and conscious and non-conscious bias all contribute to promotion inequity. Because salary is dependent on academic rank in many institutions, this disparity ensures that URMM faculty are paid less than their peers [16].

**Table 1:** **Promotion rates for black, Latino and white faculty**

Study author and year	Assistant to Associate			Associate to Full		
	Black	Latino	White	Black	Latino	White
Nunez-Smith et al. (2012) [21]	21.7%	26.2%	30%	18.8%	23.5%	30.2%
Fang et al. (2000) [19]	URM* 30%		46%	URM 36%		50%

*URM refers to black, Mexican American, Mainland Puerto Rican and Native American faculty members.

## Summary

**Figure 1:**
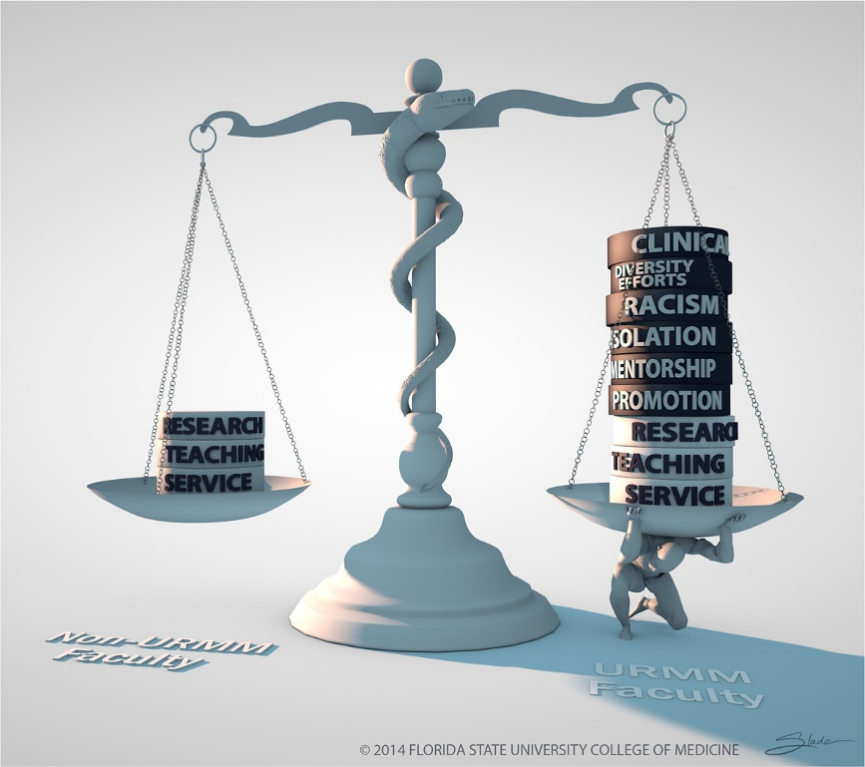
**Additive effect of the minority tax.**

The sum of these disparities presents a considerable barrier to success for URMM faculty, as illustrated in Figure 1.

The inequities described make it very difficult for them to remain and advance in academic medicine. It is also a regressive disparity—URMM faculty that have “extra” responsibilities are more likely to be found in the lowest paying ranks.

The relative absence of URMM faculty and especially in leadership roles has a negative effect on all medical students and house staff, but particularly URMM trainees. These negative effects include: less research regarding the health care needs of minority patients, limited exposure to underserved populations, and fewer mentors for URMM students. URMM faculty are essential to pipeline programs, and they provide support for URMM students in the form of role models, educators and mentors [2]. Since URMM faculty teach students to care for underserved/minority patients by caring for those patients themselves, their relative absence among the faculty also has a negative effect on patient care. As the US population becomes more diverse, educators need to ensure that our physician workforce is willing and committed to caring for diverse patients. Increasing the numbers of URMM faculty is an important part of that effort. Since URMM faculty are also more likely to engage in health disparities research than their non-URMM counterparts [5], increasing the proportion of faculty from URMM groups would also benefit the nation’s research agenda to eliminate health care disparities.

### Addressing the URMM responsibility disparity

We can learn of effective ways to eliminate these disparities affecting URMM faculty from our colleagues in human resources. Among possible interventions are:

1. Value diversity effort fairly [22]
  - Recognize that the URMM responsibility disparity exists and adjust assignment of responsibilities accordingly
  - Work to ensure that clinical and community endeavors are counted toward promotion
  - Assign promotion value to work in the area of diversity
2. Employ rules that are in harmony with the institution’s stated service goals and mission [22]
  - Increase awareness and avoidance of mission drift, i.e. institutional departure from the service mission [18]
  - Fund stated institutional diversity commitments.
3. Eliminate all forms of discrimination
  - Institute policies and procedures that address and correct bias [6]
    i. Move beyond compliance with the Americans with Disabilities Act and Title VII to establish robust accountability systems for acts of discrimination by including it in annual evaluations.
  - Facilitate and support relationship formation among faculty, administrators, and learners [23].
  - Encourage positive curiosity when encountering “otherness” and recognize differences in faculty as benefitting our institutions [23].
    i. Seek training in unconscious bias for all faculty to help recognize its role in discrimination.
4. Ensure clear, frank, honest communication between administration and faculty to avoid faculty discouragement.
  - Develop transparent communication in the promotion and tenure process.
  - Develop opportunities for explicit conversations (i.e. professionally moderated retreats) about personal values to amplify the meaning faculty find in the practice of medicine and in their careers [23].
5. Develop an employee retention strategy [22]
  - This could take the form of faculty development that focuses on:
    i. Institutional culture
    ii. Networking
    iii. Professional skill development
      1. Understanding the prevalence and acceptance of unconscious bias
      2. Teaching acceptable institution specific behaviors to address silent racism.
      3. Dealing with micro-aggressions and stereotype threat [24]
      4. Avoiding isolation and marginalization
    iv. Mentoring [13]
6. Develop and implement organizational culture-change activities in medical schools involving broad participation to provide the experience (for faculty *and* leadership) of learning and collaborating in an inclusive and humanistic culture [25,26].

The authors propose that these changes could make a career in academic medicine much more attractive to URMM faculty, and can, in effect, begin to alleviate the URMM faculty responsibility disparity, and make academic medicine a more equitable career choice for graduating URMM residents. Similarly to repealing taxes, addressing these disparities requires political stamina, negotiation and coalition building. It requires a champion in the ruling body who is willing to use political capital to establish faculty equity. Diversifying academic leadership can help alleviate the URMM responsibility disparity. In addition to the cited proven institutional change programs, further research on interventions to address the URMM faculty responsibility disparity is necessary to evaluate their effectiveness. These interventions once implemented, can hopefully help create a healthy, diverse, and inclusive environment that will benefit all members of the academic community and improve health care.

### Ethics

The Human Subjects Committee at the Florida State University Institutional Review Board (IRB) does not require ethics approval for manuscripts using data that has already been published. Since this paper only uses that type of data, it is exempt from IRB review.

## Abbreviation

URMM: Underrepresented Minorities in Medicine
